# Pleomorphic Adenoma of the Cheek: A Case Report

**DOI:** 10.7759/cureus.5312

**Published:** 2019-08-03

**Authors:** Senthilnathan Periasamy, Ashok Manoharan, Himani Garg, Santhosh P Kumar

**Affiliations:** 1 Oral and Maxillofacial Surgery, Apollo Main Hospitals, Chennai, IND; 2 Oral and Maxillofacial Surgery, Saveetha Dental College and Hospital, Saveetha University, Chennai, IND

**Keywords:** pleomorphic adenoma, minor salivary gland tumour, cheek, buccal mucosa

## Abstract

Pleomorphic adenoma (PA) is the most common benign tumor affecting both major and minor salivary glands. Parotid gland is the most commonly affected major salivary gland. Among minor salivary glands, palate is the most commonly affected site followed by lips, cheeks, gingiva, floor of the mouth, and tongue. PA of buccal minor salivary glands is a very rare occurrence both in adults and children. In this report, we present a case of PA of buccal minor salivary gland in an adult patient who was successfully treated by wide local surgical excision, and after a follow-up period of one year there was no recurrence. A review of literature of PA of cheek is also presented.

## Introduction

Salivary gland tumours account for 3% of the head and neck tumours [1]. Pleomorphic adenoma (PA) is the most common salivary gland tumor, accounting for about 40%-70% of all major and minor salivary gland tumors (MGST) [2]. PA is a benign mixed tumor composed of epithelial and myoepithelial cells arranged in various morphological patterns, demarcated from the surrounding tissues by a fibrous capsule [3]. Parotid gland is the most commonly affected major salivary gland. Among the minor salivary glands, palate is the commonly involved site, with nearly 60% arising from this location. The lips, cheek, and gingiva are rare sites of occurrence [4]. In this report, we present a case of PA of buccal minor salivary gland in an adult patient which was successfully treated by wide local surgical excision.

## Case presentation

A 31-year-old female patient reported to the department of oral and maxillofacial surgery at Apollo main hospital in Chennai, Tamil Nadu, India. The patient’s chief complaint was swelling over right side of face, for the past four years. History revealed the swelling was painless, and initially smaller in size which gradually increased to the present size. She did not have any difficulty with speech and deglutition. The patient had taken homeopathic medication for the swelling for the past two years but it did not reduce in size. The patient’s past medical and dental history was not significant. On systemic examination, the patient was healthy and there was no regional lymphadenopathy.

**Figure 1:**
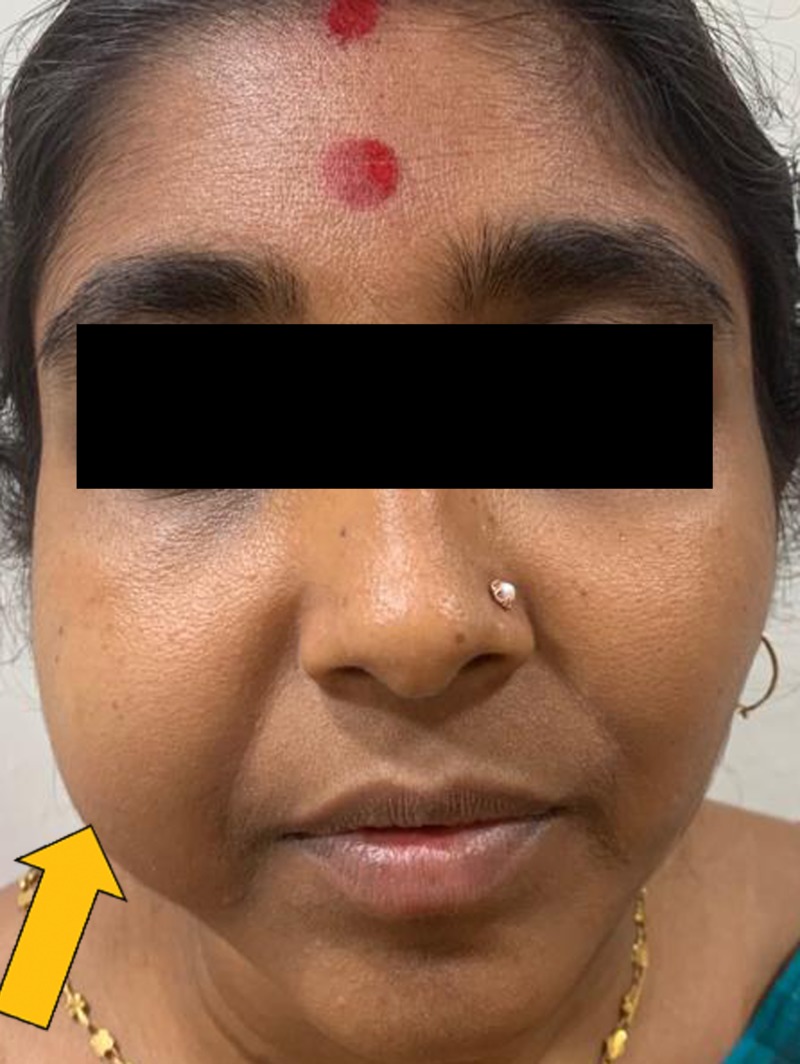
Extraoral view showing swelling in the right cheek region.

On extraoral examination, facial asymmetry was present due to swelling on the right side. A solitary dome-shaped, oval swelling with smooth surface was present on the right cheek region (Figure 1). Overlying skin was normal. Swelling was approximately in mid-cheek region measuring about 5 cm × 3 cm, extending superior-inferiorly from ala-tragus line to the lower border of mandible. Antero-posteriorly it was extending 1 cm from the right angle of mouth up to 1 cm short of right pterygomandibular raphe. On palpation the swelling was sessile, firm in consistency, nontender, nonfluctuant, nonreducible, nonpulsatile, and mobile in all planes with well-defined margins. 

**Figure 2:**
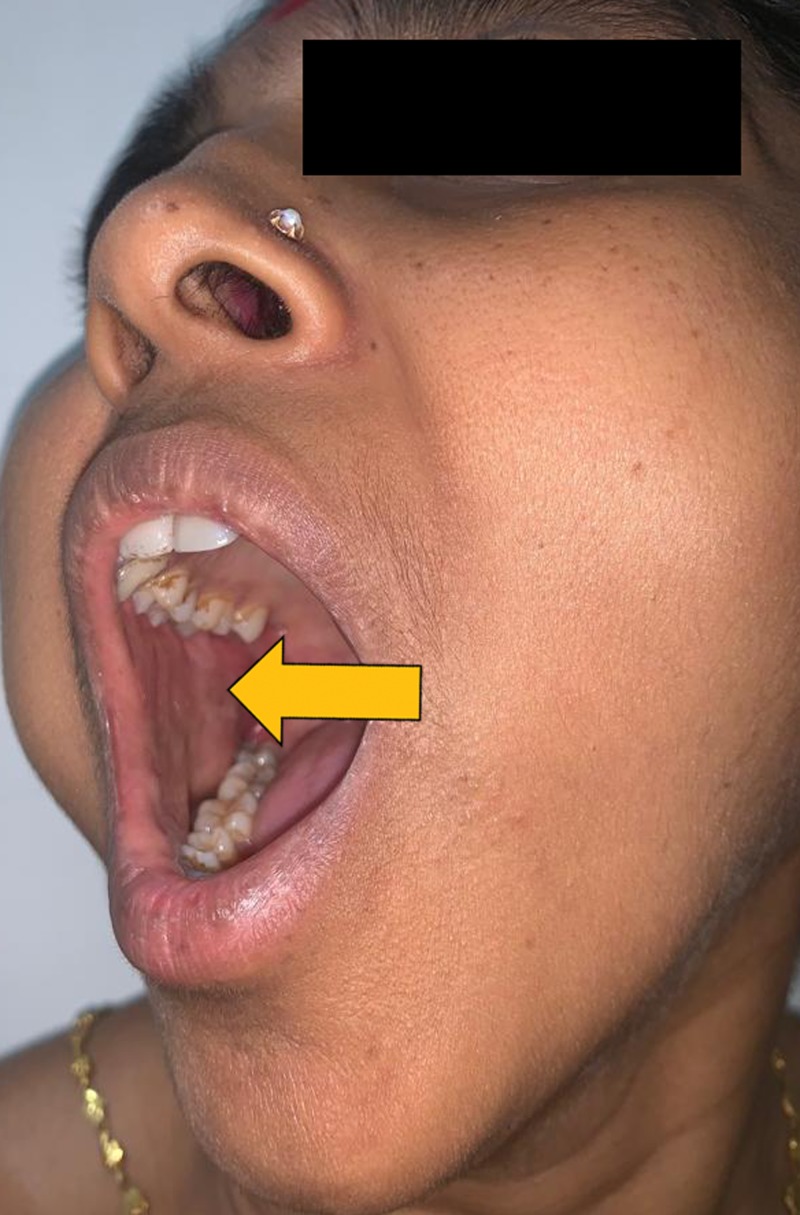
Intraoral view reveals intact healthy mucosa over the mass.

Intraoral examination revealed no significant findings. The swelling was covered by intact healthy mucosa (Figure 2). On bimanual palpation the mass could be felt between buccal mucosa and skin and it was not fixed to the deeper structures. Mouth opening was adequate and there were no signs of motor or neurosensory deficit in the region of the lesion. 

**Figure 3:**
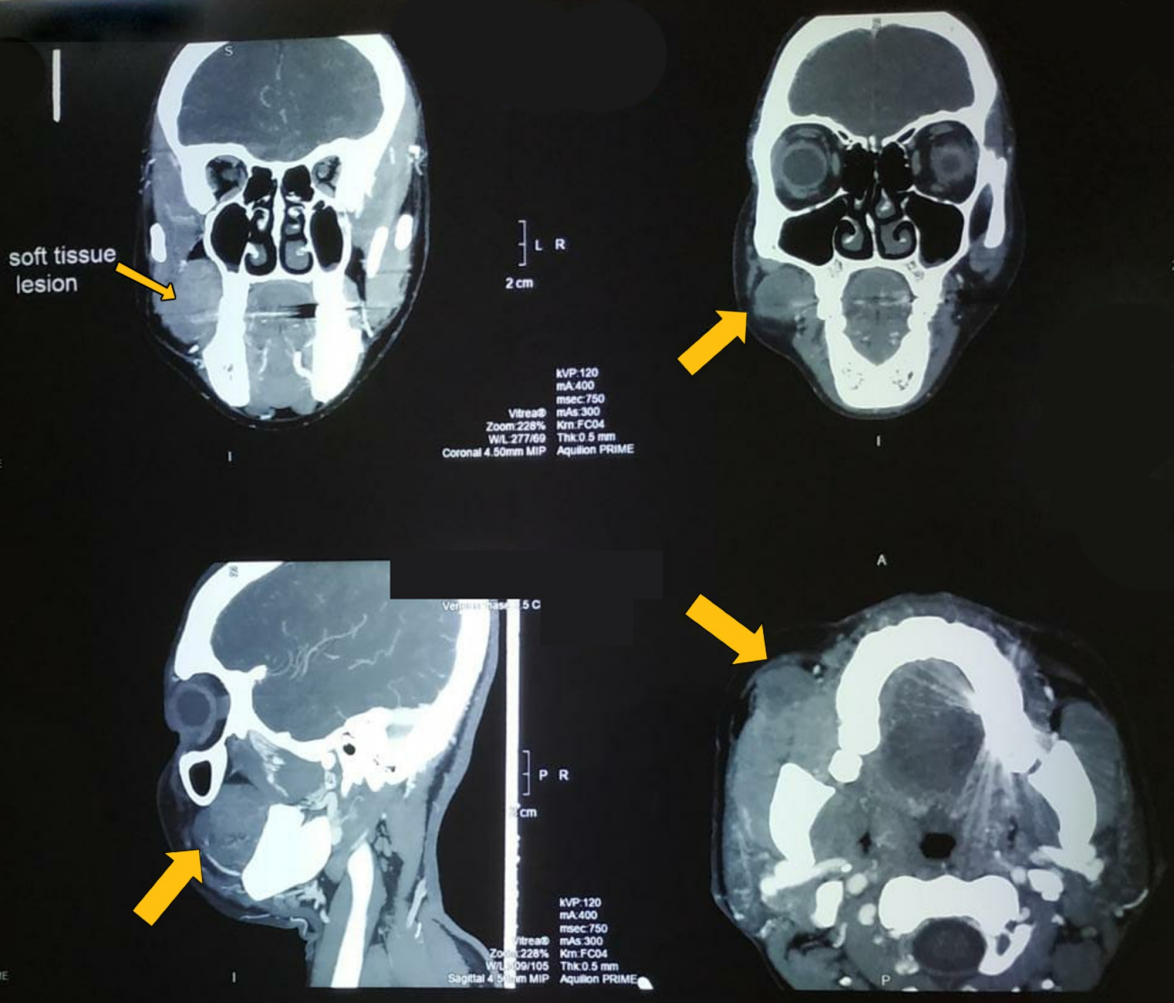
CT scan sections showing well-defined homogeneously enhancing lesion in the right buccal space region.

Patient’s blood investigations were within normal limits. CT scan of face was suggestive of well-defined encapsulated homogeneously enhancing lesion in the right buccal space region without invasion of the adjacent structures (Figure 3). Differential diagnosis of the lesion included MGST, tumor of accesory parotid salivary gland, lipoma, myofibroma, and neurofibroma. Based on the history, clinical presentation and radiological investigations, the decision was made to surgically excise the lesion under general anesthesia.

**Figure 4:**
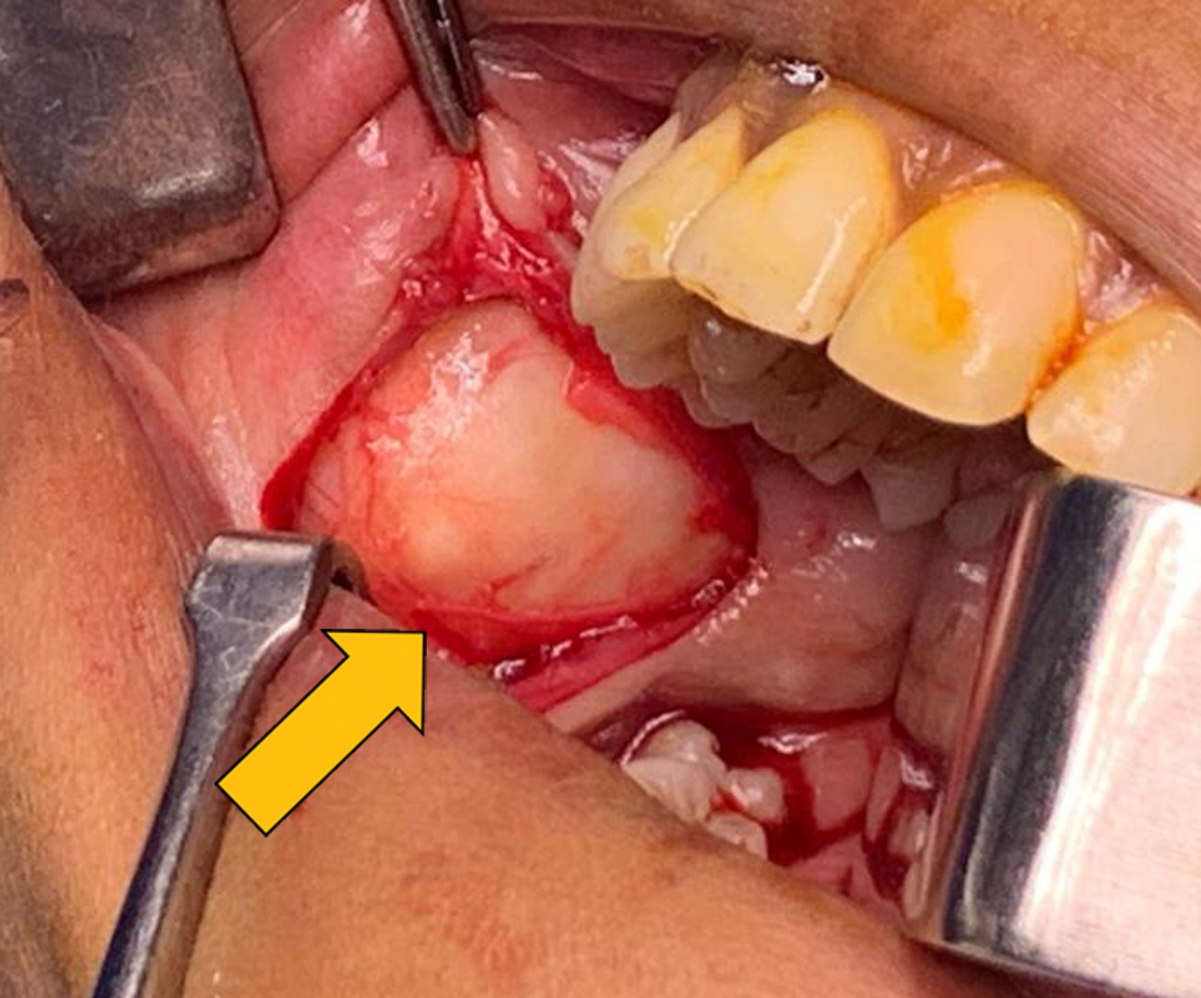
Exposure of the mass.

**Figure 5:**
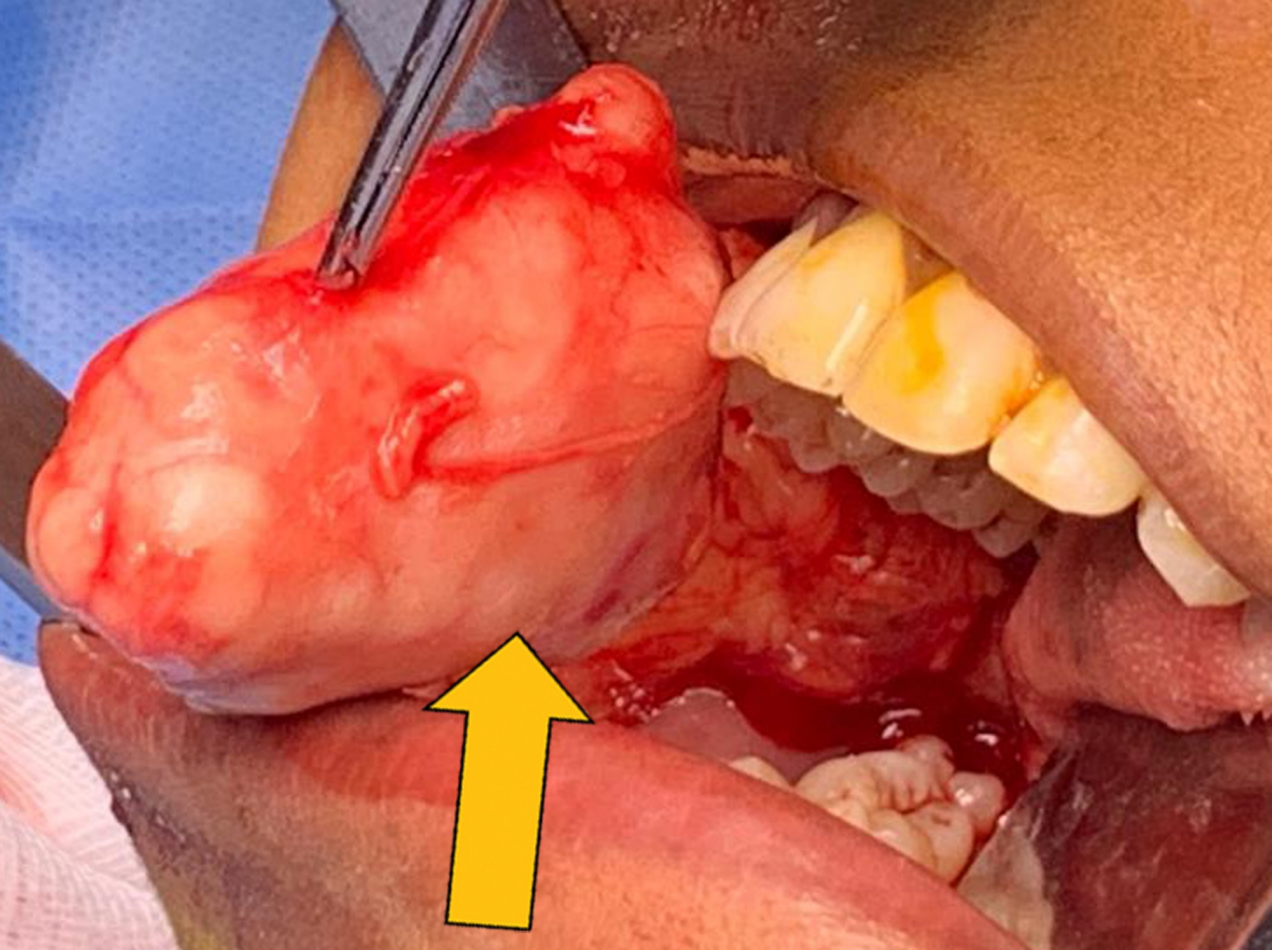
Lesion freed from the surrounding tissues.

**Figure 6:**
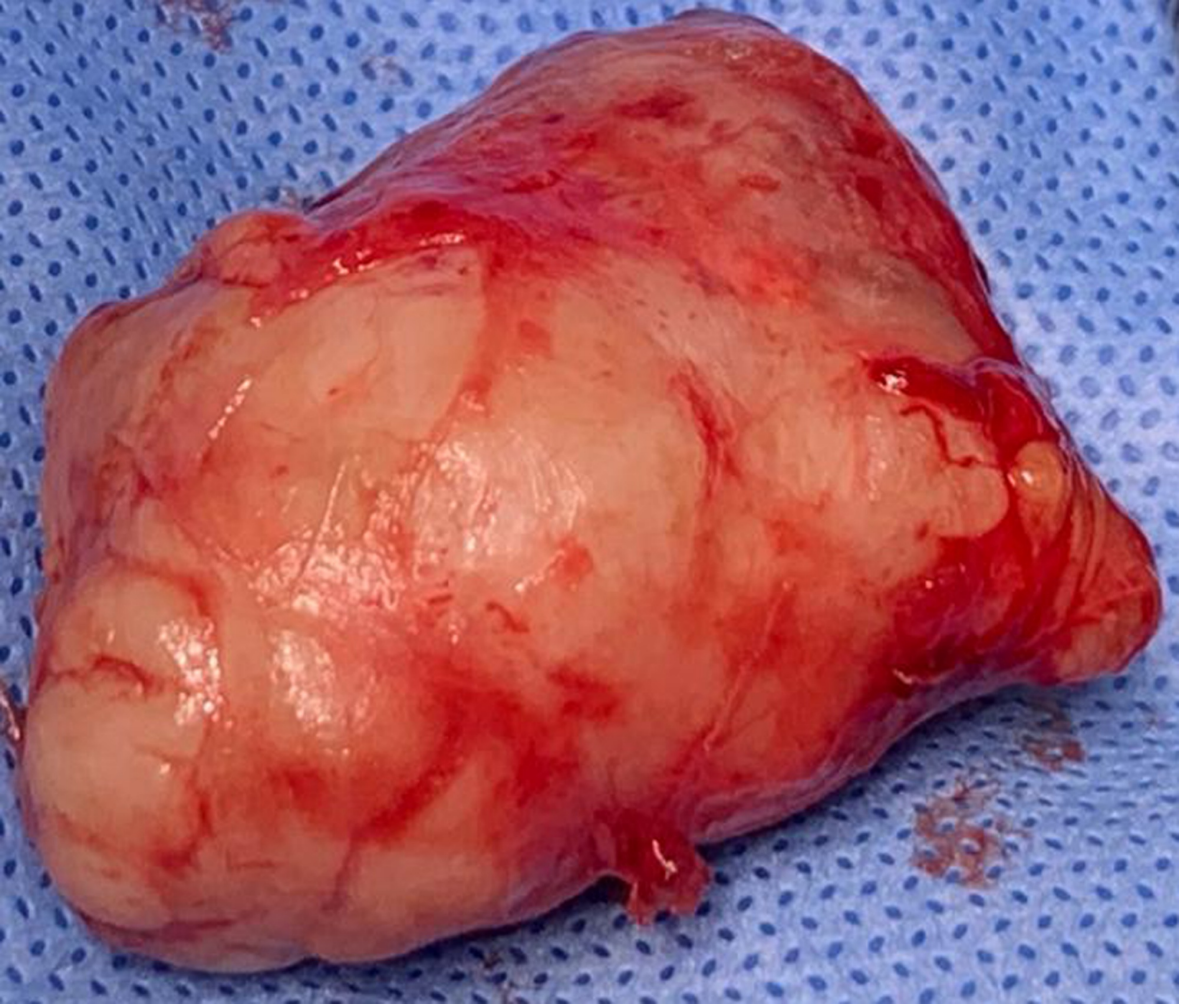
Excised specimen.

A horizontal incision was made in the right buccal mucosa parallel to the occlusal plane without injuring the parotid gland duct. Blunt dissection was done to expose the mass which was present between buccal mucosa and the buccinator muscle (Figure 4). It was freed from the surrounding tissues and the lesion was excised along with adequate margin of normal tissue (Figure 5). Hemostasis was achieved and the wound was sutured. Postoperative recovery and wound healing was uneventful. Excised specimen was sent for histopathological examination (Figure 6).

**Figure 7:**
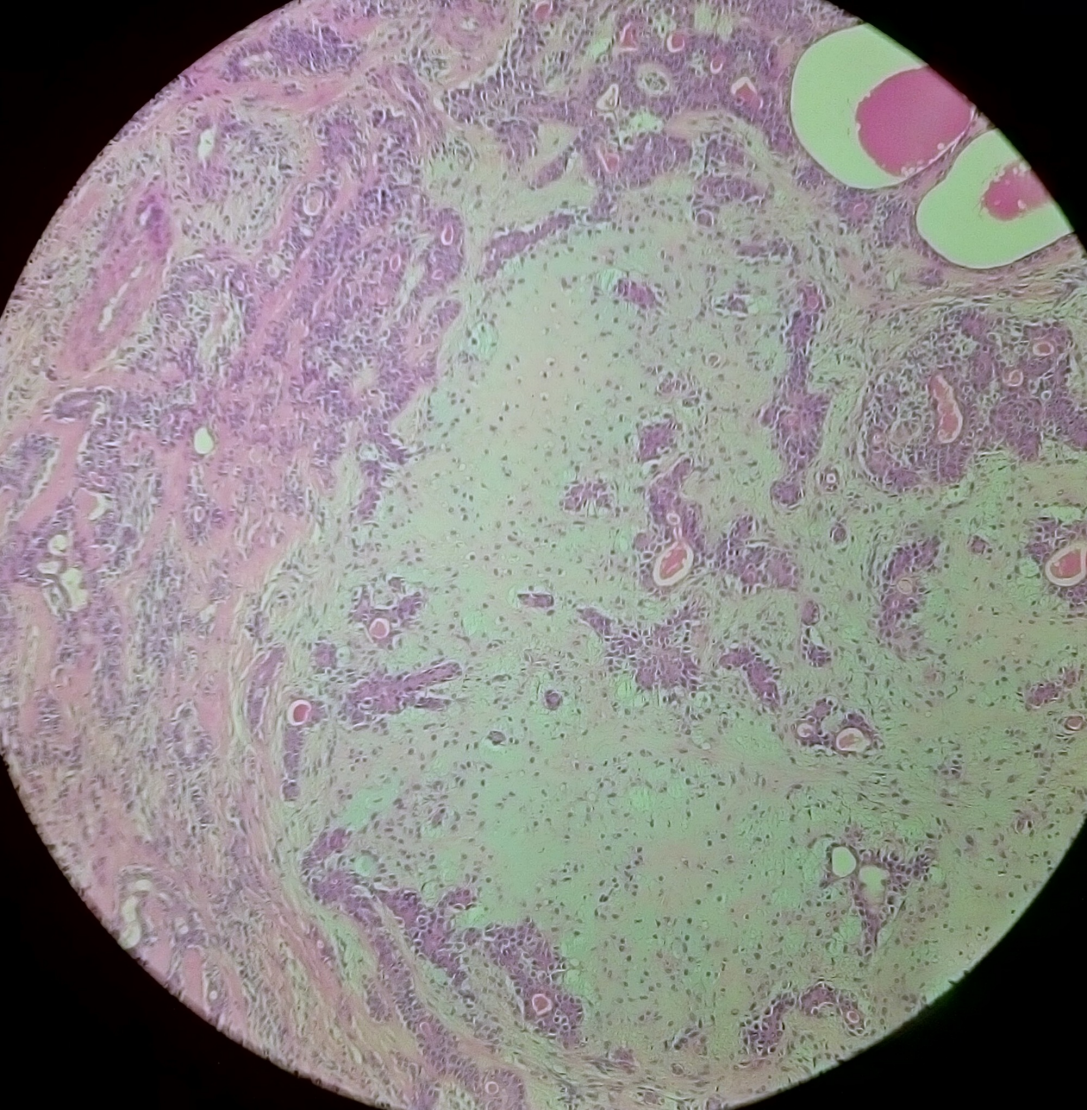
Histopathological image showing epithelial cells with myxoid and myxochondroid connective tissue stroma.

**Figure 8:**
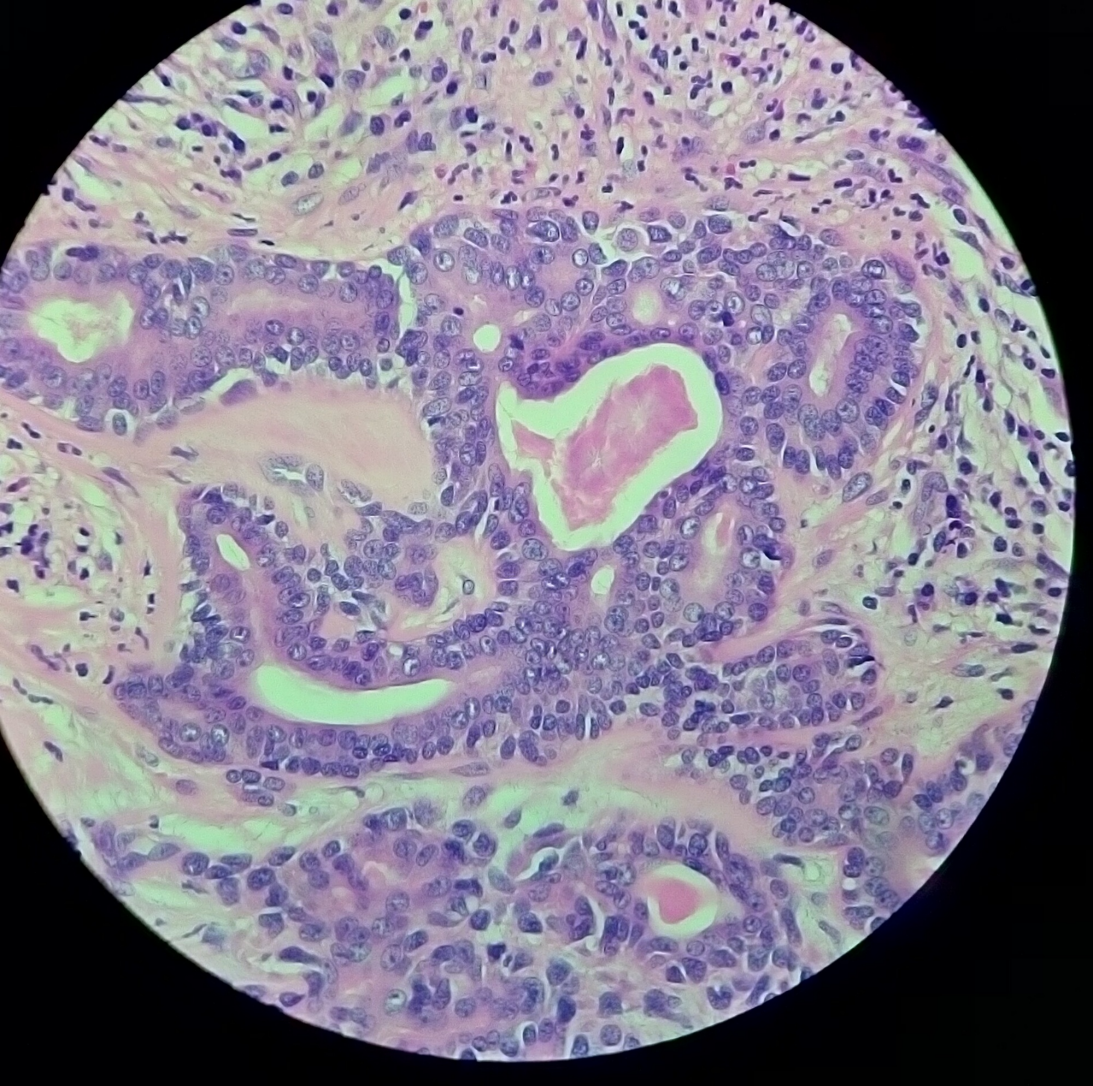
Histopathological image showing cytoarchitectural features typical of pleomorphic adenoma.

Excisional biopsy specimen revealed a single soft circumscribed encapsulated nodule, measuring 4.5 cm x 3 cm x 1.5 cm in size, grayish in color, and was soft to firm in consistency with rough surface texture. Cut section was firm and exhibited grayish tan with some gelatinous areas and foci of calcification. Histopathology report revealed an encapsulated mass composed of tubules, clusters, and anastamosing trabeculae of epithelial cells with foci of myxoid and myxochondroid areas. Cystic spaces were filled with keratin. Eosinophilic secretions were noted in the tubules (Figure 7). No significant atypia or increase in mitosis was noted. Lymphocytic cell infiltration was seen at periphery. Foci of cystic change was present (Figure 8). This confirmed the diagnosis of PA of salivary gland. Because the tumor was not associated with the parotid duct or gland, it was considered to be of buccal minor salivary gland origin. The patient is under periodic review, and there is no evidence of recurrence after one year of follow-up. 

## Discussion

Minor salivary gland tumors constitute for 22% of all salivary gland neoplasms. Majority of them are malignant with only 18% being benign [5]. Waldron et al. reported that 53%-65% of the MSGT are benign [6]. PAs accounts for the majority of benign salivary gland neoplasms. The most common site of PA of the minor salivary gland is the palate followed by lip, buccal mucosa, floor of mouth, tongue, tonsil, pharynx, retromolar area, and nasal cavity [5-7]. They are more common in adults than in children. It usually occurs in the fourth to sixth decades of life and is found more commonly in women than in men [5, 8]. Several studies have depicted varied frequency of occurrence of PA of minor salivary glands of the cheek [2, 4, 8-14] (Table 1).

**Table 1:** Literature review of the frequency of pleomorphic adenoma of minor salivary glands of the cheek.

Studies	Total number of pleomorphic adenoma cases	Number of cases in cheek	Percentage (%) of cases in cheek
Isacsson and Shear[9]	140	7	5
Fine et al.[10]	25	4	16
Chaudhry et al.[8]	476	38	8
Main et al.[11]	31	5	16
Cohen[4]	144	10	7
Bablani et al.[12]	24	4	17
Buchner et al.[13]	149	19	13
Lopes et al.[2]	65	0	0
Yih et al[14]	93	16	17

The PA of the cheek usually presents as a mobile slowly growing, painless, firm, lobulated submucosal swelling that does not cause ulceration of the overlying mucosa. These benign neoplasms are usually well circumscribed and round or oval in shape. They vary in consistency from soft and fluctuant to firm and rubbery, depending on the presence of cystic or mucoid degeneration or the formation of chondroid or osteoid tissues [8]. The size of the tumors range from 1 to 7 cm in diameter with some PA of cheek may attain larger sizes. PA of minor salivary glands are detected and treated earlier than that of major salivary glands [4]. In our case the tumor was allowed to grow to a considerate size as it did not interfere with deglutition and speech. The noticeable cosmetic deformity prompted the patient to seek treatment.

CT scan, MRI, and ultrasonography are useful in determining the size and extent of lesions and in determining the bone involvement [15]. Incisional biopsy of PA in situ may predispose to recurrence and it is contraindicated. Fine needle aspiration cytology is the preferred diagnostic modality.

The PA has three histological subtypes: myxoid (80% stroma), cellular (myoepithelial cells predominating), and mixed (classic). Histologically, they have epithelial and mesenchymal elements. Epithelial cells are arranged in cord-like and duct-like cell patterns, along with areas of epidermoid metaplasia. The intercellular matrix shows fibrous, hyaline, myxoid, cartilaginous, and osseous areas. Myoepithelial cells are responsible for such pleomorphic extracellular matrix production. In the minor glands, lesions are often more solid or cellular than those seen in the major glands, and the myoepithelial cells are often polygonal with a pale eosinophilic cytoplasm giving an epithelioid or plasmacytoid phenotype [16]. In our case histopathology report confirmed the diagnosis of PA.

Differential diagnosis for the mass in the cheek includes: MGST, tumor of accessory partid salivary gland, lipoma, myofibroma, neurofibroma, sebaceous cyst, epidermoid cyst, dermoid cyst, mucoepidermoid carcinoma, and adenoid cystic carcinoma [3-4].

Surgical excision with an adequate margin of normal surrounding tissue is the treatment of choice for PA of the cheek [4]. In our case the lesion was encapsulated, situated between the cheek mucosa and the buccinator muscle which was highly suggestive of a benign tumor of minor salivary gland origin. The lesion was excised intact with its capsule. Ra­diotherapy is not indicated due to the radioresistant nature of the tumor. Inadequate resection, rupture of the capsule, or tumor spillage during excision can lead to local recurrence as these tumors often have microscopic pseudopod-like extensions into the surrounding tissues through the capsule [17]. Spiro et al. reported a recurrence in 7% of 1,342 patients with benign parotid neoplasms, and in 6% of patients with benign MGSTs [5]. In few cases PA of minor salivary glands can undergo malignant transformation into carcinoma for example PA and metastasizing benign mixed tumor. Recurrence after many years of surgical excision as well as malignant transformation is a concern, hence long-term follow-up of up to 10 years is necessary [4].

## Conclusions

The varied presentation of a MGST makes the diagnosis challenging even for an experienced surgeon. PA of the cheek is a rare neoplasm and therefore its diagnosis requires a high index of suspicion. PA should be considered in the differential diagnosis of cheek masses both in young and adult patients. Complete wide local surgical excision is the treatment of choice. Patients should be followed up for a longer period of time due to the possibility of late recurrences.
